# Mental Health Outcomes in US Children and Adolescents Born Prematurely or with Low Birthweight

**DOI:** 10.1155/2013/570743

**Published:** 2013-11-12

**Authors:** Gopal K. Singh, Mary Kay Kenney, Reem M. Ghandour, Michael D. Kogan, Michael C. Lu

**Affiliations:** US Department of Health and Human Services, Health Resources and Services Administration, Maternal and Child Health Bureau, 5600 Fishers Lane, Room 18-41, Rockville, MD 20857, USA

## Abstract

We examined the effects of prematurity (<37 weeks of gestation) and low birthweight (<2500 g) on mental health outcomes among US children aged 2–17 years. The 2011-2012 National Survey of Children's Health (*N* = 95,677) was used to estimate prevalence of parent-reported mental health problems in children. Prevalence of mental disorders was 22.9% among children born prematurely, 28.7% among very-low-birth-weight (<1500 g) children, and 18.9% among moderately low-birth-weight (1500–2499 g) children, compared with 15.5% in the general child population. Compared to those born full term, children born prematurely had 61% higher adjusted odds of serious emotional/behavioral problems, 33% higher odds of depression, and 58% higher odds of anxiety. Children born prematurely had 2.3 times higher odds of autism/ASD, 2.9 times higher odds of development delay, and 2.7 times higher odds of intellectual disability than term children. Very-low-birth-weight children had 3.2 times higher odds of autism/ASD, 1.7 times higher odds of ADD/ADHD, 5.4 times higher odds of development delay, and 4.4 times higher odds of intellectual disability than normal-birth-weight children. Social factors were significant predictors of mental disorders in both premature/low-birth-weight and term/normal-birth-weight children. Neurodevelopmental conditions accounted for the relationship between prematurity and depression/anxiety/conduct problems. Prematurity and low birthweight are significant risk factors for mental health problems among children.

## 1. Introduction

Perinatal factors such as preterm birth and low birthweight (LBW) have significant impacts on the health and well-being of children and adolescents, which have been shown to persist well into adulthood and, indeed, throughout the life course [1–4]. Prematurity/LBW has long been the second leading cause of infant death in the United States after congenital malformations [5]. While US infant mortality rates due to prematurity and LBW (<2500 grams) have declined markedly over time, preterm (<37 weeks of gestation) infants are still 15 times more likely to die before their birthday than those born at term [5, 6]. Nearly two-thirds of all infant deaths in the USA occur among infants who are born preterm [5].

Nearly a quarter of all infants born with very low birthweight (<1500 grams) die during the first year of life [5]. The infant mortality rates for very-low-birth-weight (<1500 grams) and moderately low-birth-weight (1500–2499 grams) infants are, respectively, 105 and 6 times higher than the rate for normal-birth-weight infants [5, 6]. Despite the marked improvements in survival rates, preterm and LBW infants remain vulnerable to many physical and mental health problems, including respiratory distress syndrome, asthma and lung disease, cerebral palsy, visual, hearing, and cognitive impairments, and a range of behavioral and developmental disorders [1, 2, 4]. While physical health problems among premature and LBW children are well-documented, behavioral and mental health problems among them are less well studied [1]. Studies that do exist have reported increased risks of depression, anxiety, behavioral, and conduct disorders among preterm and LBW children, compared with the general population or children who are born at full term or with normal birthweight [2, 7–18]. Studies have also reported 2-to-4-fold higher rates of more serious neurodevelopmental conditions in premature/LBW populations, including autism spectrum disorders (ASD), ADHD, and learning difficulties [2, 19–23].

Most of the studies that have examined mental health problems among children born preterm or with LBW are small case-control studies, the findings of which may not be generalizable to the population at large or the US population [2, 7–23]. Moreover, such studies tend to assess mental health outcomes associated with prematurity/LBW at a specific age during late childhood or adolescence [2, 7–23]. Mental health assessment among preterm/LBW children during the entire age range, extending from early childhood to late adolescence, remains unexplored. Although several studies have focused on specific mental disorders, few studies have examined the association between prematurity/LBW and a wide range of mental health outcomes among both younger children and older adolescents in a comprehensive manner.

The 2011-2012 National Survey of Children's Health (NSCH), which is a large nationally representative sample of US children aged 0–17 years, allows us to explore the association between perinatal conditions and mental health outcomes in both childhood and adolescence, while controlling for current household socioeconomic status (SES), family environments, and other demographic variables. In this study, we examine (1) the prevalence of overall and specific mental health outcomes among US children by prematurity and LBW before and after controlling for household SES and sociodemographic characteristics, (2) whether mental health outcomes associated with prematurity and LBW vary by child's sex and age, (3) whether social factors are significant predictors of mental health problems in both preterm/LBW and general child populations, and (4) the extent to which neurodevelopmental conditions such as autism/ASD, ADHD, and developmental delay might account for the relationship between perinatal conditions and common emotional/behavioral disorders of depression, anxiety, and conduct problems.

## 2. Methods

Data for the present study came from the 2011-2012 NSCH [22, 23]. The survey was conducted by the National Center for Health Statistics (NCHS), with funding and direction from the Maternal and Child Health Bureau [24, 25]. The purpose of the NSCH was to provide national and state-specific prevalence estimates for a variety of children's health and well-being indicators [24, 25]. The survey included an extensive array of questions about children's health and the family, including parental health, stress and coping behaviors, family activities, and parental concerns about their children [24, 25]. Interviews were conducted with parents, and special emphasis was placed on factors related to children's well-being.

The 2011-2012 NSCH was a cross-sectional telephone survey conducted between February 2011 and June 2012 [24, 25]. The two previous rounds of the NSCH were conducted in 2003-2004 and 2007-2008 [25–28]. The 2011-2012 survey had a sample size of 95,677 children <18 years of age, including a sample of >1,800 children per state [24, 25]. In the survey, a random-digit-dial sample of households with children <18 years of age was selected from each of the 50 states and the District of Columbia. One child was selected from all children in each identified household to be the subject of the survey [24, 25]. Interviews were conducted in English, Spanish, and four Asian languages. The respondent was the parent or guardian who knew most about the child's health status and health care. All survey data were based on parental reports. The interview completion rate for the 2011-2012 NSCH, a measure of the response rate indicating the percentage of completed interviews among known households with children, was 54.1% for the landline sample and 41.2% for the cell phone sample [24, 25]. Substantive and methodological details of the 2011-2012 NSCH are described elsewhere [24]. The NCHS Research Ethics Review Board approved all data collection procedures for the survey.

**Figure 1:**
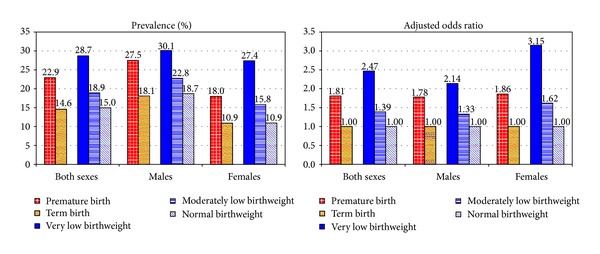
Prevalence (%) and adjusted odds of mental disorders (one or more of the eight conditions) among US children aged 3–17 years by prematurity and low birthweight. *Notes*. Differences in prevalence and odds between preterm and birthweight categories were all statistically significant at *P* < 0.001. Odds ratios were adjusted for child's age, sex, race/ethnicity, household composition, race/ethnicity, household composition, place of residence, household poverty, and education levels. Term birth and normal birthweight were the respective reference categories for the prematurity and birthweight variables. *Source*. The 2011-2012 National Survey of Children's Health (*N* = 80,649).

The sample size for the present analysis was 85,535 children aged 2–17 years. Each of the 10 mental health outcomes studied is defined in Table 1. Eight specific mental health conditions were considered, with depression, anxiety, and conduct disorders combined into a composite emotional/behavioral problem variable. Additionally, we combined all eight disorders, including depression, anxiety, conduct disorders, autism/ASD, ADD/ADHD, developmental delay, learning disability, and intellectual disability, to define the total prevalence of mental disorders among children (Figure 1). Premature birth and birthweight were the primary covariates of interest. Premature birth is defined as a delivery before 37 completed weeks of gestation [5]. In the survey, prematurity was determined by asking the parents if their child was born prematurely, that is, more than 3 weeks before his/her due date [24]. Parents reported children's birthweight retrospectively. Children with birthweight <53 oz (<1,500 g) were classified as very-low-birth-weight (VLBW) children and those with birthweight between 53 and 88 oz (1500–2499) were classified as moderately low-birth-weight (MLBW) children. Children with birthweight <89 oz (<2500 g) were defined as low-birth-weight (LBW) children and those with ≥89 oz were considered as normal-birth-weight (NBW) children [5].

Using a life-course/social-determinants-of-health framework and past research as a guide, we considered, in addition to prematurity and birthweight, the following covariates of childhood mental disorders: child's age, sex, race/ethnicity, household composition, metropolitan/nonmetropolitan residence, household/parental education level, and household poverty status measured as a ratio of family income to the poverty threshold [25, 29–35]. These covariates were measured as shown in Tables 2–6.

Income was imputed for 9% of the observations by using a multiple imputation technique [24, 27]. Less than 1% of the observations had missing data on prematurity and 4.8% of the observations had missing data on birthweight. For all other covariates, there were few or no missing cases, which were excluded from the multivariate analyses, yielding an effective sample size of 84,685 for most of the analyses.

The *χ* ^2^ statistic was used to test the overall association between covariates and mental disorders. The *t*-statistic was used to test the difference in prevalence between any two groups. Logistic regression was used to examine the association between perinatal factors and mental health problems, after adjusting for the above covariates. To account for the complex sample design of the NSCH, SUDAAN software was used to conduct all statistical analyses [36].

## 3. Results

### 3.1. Descriptive Characteristics of the Study Sample

Descriptive characteristics of the study sample are shown in Table 2. Approximately 11.5% of the children aged 2–17 were born prematurely and 9.5% with LBW. Non-Hispanic white children were the largest racial/ethnic group (51.6%), followed by Hispanics (23.6%) and blacks (13.4%). Approximately 22.0% of children lived below the poverty line, and 11.6% of children had parents who had less than a high school education. Nearly one-fifth of children lived in single-mother households and 9.6% of children lived in stepfamily households.

### 3.2. Inequalities in Prevalence and Odds of Overall Mental Health Problems

The prevalence of mental health problems was higher in premature/LBW children than in term/normal-birth-weight children (Figure 1). The prevalence of mental disorders was 22.9% among children born prematurely, 28.7% among VLBW children, and 18.9% among MLBW children, all significantly higher than the prevalence for term/normal-birth-weight children (<15%) or for the general child population (15.5%) (Figure 1). The prevalence of mental disorders was much higher among prematurely born male than female children (27.5% versus 18.0%). Relative risks of mental disorders were largely unchanged after adjusting for socioeconomic and demographic covariates. Preterm children had 82% higher adjusted odds of mental disorders (OR = 1.81; 95% CI = 1.61−2.04) than term children. Male VLBW children had 2.1 times higher adjusted odds (OR = 2.14; 95% CI = 1.39−3.28) and female VLBW children 3.2 times higher odds (OR = 3.15; 95% CI = 2.05−4.85) than their normal-birth-weight counterparts (Figure 1).

### 3.3. Inequalities in Prevalence and Odds of Specific Mental Disorders

Tables 3 and 4 show prevalence of specific mental health problems according to perinatal and sociodemographic characteristics. Approximately 8.1% of children born prematurely had mental/emotional problems (depression, anxiety, or conduct disorders), compared with 5.7% of term children (Table 3). While prematurity and birthweight differentials in depression were not statistically significant, preterm children had significantly higher prevalence of anxiety (4.7%) and conduct disorder (4.5%) than term children. Both prematurity and birthweight were strongly associated with the prevalence of ASD and other neurobehavioral conditions (Table 4). Approximately 3.6% of premature children were diagnosed with autism/ASD, compared with 1.7% of term children. There was a birthweight gradient in the ASD prevalence, with 4.5% of VLBW, 2.3% of MLBW, and 1.8% of NBW children being diagnosed with ASD. Nearly 11% of VLBW or premature children had ADD/ADHD, compared with <8% of term/NBW children. The prevalence of development delay was 3–5 times higher among premature/VLBW children than that among term/NBW children. The prevalence rates of learning disability and intellectual disability among premature/LBW children were about two times higher than those among term/NBW children. More than 18% of VLBW children had learning disability, and 3.9% of VLBW children were diagnosed with intellectual disability (Table 4).

Adjustment for socioeconomic and demographic covariate did not attenuate the effects of prematurity and LBW on specific mental disorders. Compared to term children, children born prematurely had 61% higher adjusted odds of serious emotional/behavioral problems, 33% higher odds of depression, and 58% higher odds of anxiety (Table 5). Children born prematurely had 2.3 times higher odds of autism/ASD, 1.5 times higher odds of ADD/ADHD, 2.9 times higher odds of development delay, 2.1 times higher odds of learning disability, and 2.7 times higher odds of intellectual disability than those born at term (Table 6). The risk of several mental disorders increased significantly for very-low-birth-weight and moderately low-birth-weight children. VLBW children had 3.2 times higher odds of autism/ASD, 1.7 times higher odds of ADHD, 5.4 times higher odds of development delay, and 4.4 times higher odds of intellectual disability than normal-birth-weight children (Table 6). LBW was associated with increased risk of emotional/behavioral problems, particularly among female children, with the magnitude of the mental health effects being generally greater for females than males (Table 7).

### 3.4. Social Factors as Important Predictors of Mental Health Problems

SES, race/ethnicity, and household structure were significant predictors of mental health problems in the general population as well as among premature/LBW and term/normal-birth-weight children. Children living in poverty had 2.0–3.6 times higher odds of depression, anxiety, and conduct disorders than their most-affluent counterparts. Children in stepfamilies and single-mother households had 1.7–3.4 times higher odds of depression, anxiety, and conduct disorders than children from two-parent biological families. Black and Hispanic children had 46–50% lower adjusted odds of emotional/behavioral problems than non-Hispanic white children (Table 5). Although the association between social factors and neurodevelopmental conditions was less pronounced, poor children maintained 1.7–2.0 times higher adjusted odds of ADD/ADHD, intellectual disability, developmental delay, and learning disability than their affluent counterparts (Table 6). Although the income gradients in autism/ASD were not consistent, those with higher poverty levels had significantly higher risks than the most-affluent children. Non-Hispanic white children and those in single-mother households remained at significantly higher risk of all neurodevelopmental conditions even after controlling for SES (Table 6).

Social inequalities in mental health problems were smaller among premature/LBW children than among term/normal-birth-weight children (data not shown). For example, among premature/LBW children, 27.1% of children in stepfamilies and 20.2% of children in single-mother households had mental health problems, compared with 17.2% of children in two-parent biological families. Among term/normal-birth-weight children, the respective prevalence rates were 21.8%, 20.2%, and 11.3%. Among premature/LBW children, 25.0% of poor children had mental health problems compared with 17.3% of affluent children (adjusted OR = 1.53; 95% CI = 1.11–2.12), whereas among term/normal-birth-weight children, 19.2% of poor children had mental health problems compared with 11.9% of affluent children (adjusted OR = 1.97; 95% CI = 1.66–2.35).

### 3.5. Regression Models with Comorbid Conditions

We estimated regression models of both the overall emotional/behavioral problem variable and specific conditions of depression, anxiety, and conduct disorder that included sociodemographic characteristics and prematurity, along with comorbid conditions of ASD, ADD/ADHD, development delay, learning disability, and intellectual disability. The increased risks of mental health problems associated with prematurity were accounted for by these neurodevelopmental conditions, whose effects on emotional/behavioral problems were quite marked (data not shown for brevity). Children diagnosed with autism, ADHD, developmental delay, and learning disability, respectively, had 4.4, 8.4, 3.0, and 2.4 times higher adjusted odds of emotional/behavioral problems than children without such neurodevelopmental conditions. Children diagnosed with autism had 2.1, 4.7, and 3.3 times higher adjusted odds of depression, anxiety, and conduct disorders than children without autism, respectively. Children with ADD/ADHD had 5.8, 4.9, and 14.0 times higher adjusted odds of depression, anxiety, and conduct disorders than children without ADD/ADHD, respectively. In each of the comorbidity models, SES, family structure, and race/ethnicity remained strong risk factors for mental health problems.

The prevalence of mental health problems increased with child's age. Approximately 19.6% of adolescents aged 12–17 had mental health problems, compared with 15.9% of children aged 6–11 and 6.7% of children aged 3–5 years. Given that the prevalence of overall mental health problems and of depression, anxiety, ADHD, learning disability, and intellectual disability were mostly concentrated among older children and adolescents, we explored age interactions by estimating prematurity and LBW models stratified by child's age. However, the age interactions were not statistically significant, and the effects of the prematurity and LBW on mental health problems were similar for younger and older children and adolescents (data not shown).

## 4. Discussion

Although the rates of preterm birth and LBW in the USA have declined slightly in recent years, the current rates remain fairly high and represent a 10–16% increase compared to the rates in 1990 [37, 38]. Growing economic, social, and healthcare costs associated with prematurity and LBW are a major public health concern in the USA [1], and their marked impact on mental health problems during childhood and adolescence, as reported here, adds to the overall health and societal burden associated with prematurity [1]. According to our study, approximately 23% or 1.6 million premature/LBW children and adolescents in the USA are estimated to have one or more mental health problems. In all, 15.5% or an estimated 9.6 million US children aged 3–17 have mental health problems.

To our knowledge, ours is the first large-scale population-based US study to examine the prevalence of several mental health problems among preterm and LBW children using a nationally representative sample of preschool and school-aged children and adolescents. The analysis of the large NSCH sample allowed us to study in detail age- and sex-specific variations in the prevalence of some of the rarer mental health conditions such as depression, ASD, and intellectual disability among premature and LBW children. The overall prevalence of mental health problems among premature, VLBW, and MLBW children in our study generally varied from 20% to 30%, which is consistent with the prevalence estimates from other smaller-scale studies using diagnostic evaluations despite their use of extremely low-birth-weight and gestational-age categories [2].

While our study adds to the current US and international research on mental disorders associated with perinatal conditions, additional research is needed to explain the substantial effects of social factors on mental health outcomes among both premature/LBW and term/normal-birth-weight children. Consideration of additional factors such as adverse neighborhood social conditions, built environments, parental health status, parental stress, family conflict, family cohesiveness, social support, child's exposure to environmental tobacco smoke, alcohol and substance use, excess television viewing or recreational computer use, and physical inactivity may further explain the role of social factors in mental health problems [29, 31–35].

Mental health effects of prematurity and LBW reported here are generally consistent with previous research that shows higher risks of mental disorders in children associated with these perinatal outcomes [2, 7–23]. Compatible with our findings, inverse gestational-age and birth-weight gradients in ADHD and ASD have been reported previously [2, 18, 19, 22]. Additionally, we found a birthweight specific gradient in overall mental health prevalence and in development delay, learning disability, and intellectual disability.

In our study, the association between prematurity/LBW and mental health problems was largely explained by the consideration of neurodevelopmental conditions of ASD, ADHD, and developmental delay. This appears to provide evidence in support of biological pathways through which prematurity and LBW might lead to increased risks of mental health problems [1, 2]. Brain injuries and changes in brain development associated with premature births have been implicated as the causal mechanism for the increased risk of mental disorders in premature/LBW children [2]. However, even in children diagnosed with neurodevelopmental conditions, socioeconomic circumstances, family structure, and race/ethnicity played an important role in determining mental health risks, albeit to a smaller degree than in term/normal-birth-weight children.

A major strength of our study includes estimating the effects of prematurity and LBW on a variety of mental health conditions across the entire age range of childhood and adolescence. Another important contribution of this study is the estimation of overall prevalence of mental disorders in the preterm/LBW and the general child populations. The other strengths of our study include the large sample size, the generalizability of our findings, and examination of whether the effects of prematurity/LBW and socioeconomic factors vary by sex, age, and mental health outcome.

Our study has limitations. Children's mental health conditions in the NSCH were based on parental reports and may not accurately reflect the true prevalence, particularly among older adolescents [32, 33]. However, the prevalence of mental health disorders reported here is consistent with that reported in other epidemiologic studies [2, 39, 40]. Second, the prematurity and birthweight measures in the NSCH may be subject to recall bias, particularly among mothers of older children and adolescents, which may underestimate the prevalence and mental health effects of prematurity/LBW among older adolescents. However, a comparison of rates of prematurity and LBW by child's age in the NSCH and those based on vital records over time shows a substantial agreement. For example, the proportion of children aged 1 year born prematurely in the 2011-2012 NSCH was 12.2%, compared with 12.0% in the 2010 national vital records [37, 38]. Approximately 10% of children aged 17 in the NSCH were premature, compared with 11.0% in the vital records in 1994 [37, 38]. Although LBW in NSCH is not as precisely measured (in grams) as in vital records and is slightly overestimated, the correspondence in birthweight appears to be good between the two data sources. While 9.7% of children aged <1 year in the NSCH were of LBW, the rate from the vital records in 2011 was 8.1% [38]. Approximately 7.8% of children aged 17 in the NSCH were LBW, compared with 7.3% in the vital records in 1994 [37]. Third, because of the cross-sectional nature of the NSCH, causal inferences about the relationships between more contemporary measures of the social and familial environments and childhood mental health problems cannot be drawn [32, 33]. Lastly, as with most sample surveys, the potential for nonresponse bias exists for the NSCH, implying that the sample interviewed differed from the targeted child population in a systematic fashion [25, 27]. Since response rates in the NSCH tend to be lower in urban areas and low-income and ethnic-minority populations, differential nonresponse bias might affect (most likely underestimate) the impact of social factors on mental health problems [27]. However, the nonresponse adjustment to the sampling weights in the NSCH might have reduced the potential magnitude of these biases [27].

In conclusion, the evidence presented here suggests that premature birth and LBW are significant risk factors for mental health problems among children and adolescents. The most prevalent disorders among premature/LBW children are ADHD, learning disability, developmental delay, and anxiety. Increased mental health surveillance and screening are recommended for children born prematurely and/or with LBW, providing the opportunity for early diagnosis and intervention [2]. Given the important role of social factors in overall and specific mental health problems, including neurodevelopmental conditions, improvements in contemporary social environments as well as socioeconomic circumstances during pregnancy can be vital in preventing and reducing mental health problems in children.

## Figures and Tables

**Table 1:** Definitions of selected mental health indicators in the 2011-2012 National Survey of Children's Health.

Indicator	Definition
(1) Behavioral/emotional problem	Based on the questions, “Has a doctor or other health-care provider ever told you that the child had depression, anxiety, or behavioral or conduct problems, such as oppositional defiant disorder or conduct disorder? Does the child currently have depression, anxiety, or behavioral or conduct problems?” this composite, global mental health indicator is defined for children aged 2–17 years.

(2) Serious behavioral/emotional problem	This indicator is defined if the child's current depression, anxiety, and behavioral/conduct problems were described as moderate or severe. this indicator is defined for children aged 2–17 years.

(3) Depression	Based on the questions, “Has a doctor or other health-care provider ever told you that the child had depression? Does the child currently have depression?” this indicator is defined for children aged 2–17 years.

(4) Anxiety	Based on the questions, “Has a doctor or other health-care provider ever told you that the child had anxiety problems? Does the child currently have anxiety problems?” this indicator is defined for children aged 2–17 years.

(5) Oppositional defiant or conduct disorder	Based on the questions, “Has a doctor or other health-care provider ever told you that the child had behavioral or conduct problems, such as oppositional defiant disorder or conduct disorder? Does the child currently have behavioral or conduct problems?” this indicator is defined for children aged 2–17 years.

(6) Autism spectrum disorder	Based on the questions, “Has a doctor or other health-care provider ever told you that the child had autism, Asperger's Disorder, pervasive developmental disorder, or other autism spectrum disorder (ASD)? Does the child currently have autism or ASD?” this indicator is defined for children aged 3–17 years.

(7) ADD/ADHD	Based on the questions, “Has a doctor or other health-care provider ever told you that the child had attention deficit disorder (ADD) or attention deficit hyperactivity disorder (ADHD)? Does the child currently have ADD or ADHD?” this indicator is defined for children aged 2–17 years.

(8) Developmental delay	Based on the questions, “Has a doctor or other health-care provider ever told you that the child had any developmental delay that affects his/her ability to learn? Does the child currently have developmental delay problems?” this indicator is defined for children aged 2–17 years.

(9) Learning disability	Based on the questions, “Has a doctor, health-care provider, teacher, or school official ever told you that the child had a learning disability? Does the child currently have a learning disability?” this indicator is defined for children aged 3–17 years.

(10) Intellectual disability/mental retardation	Based on the questions, “Has a doctor or other health-care provider ever told you that the child had intellectual disability or mental retardation? Does the child currently have intellectual disability or mental retardation?” This indicator is defined for children aged 2–17 years.

**Table 2:** Descriptive statistics of the sample for children aged 2–17 years, according to perinatal and sociodemographic characteristics: the 2011-2012 National Survey of Children's Health (*N* = 85,535).

Sociodemographic characteristics	Unweighted number in sample	Weighted percent in sample
Child born prematurely (<37 weeks of gestation)		
Premature	9,590	11.45
Not premature	75,095	88.55
Child's birthweight		
Very low birthweight (<1,500 g)	1,255	1.68
Moderately low birthweight (1,500–2,499 g)	6,137	7.85
Normal birthweight (≥2,500 g)	73,626	90.47
Child's age (years)		
2–5	19,942	24.37
6–11	31,030	37.32
12–17	34,563	38.31
Child's sex		
Male	44,178	51.22
Female	41,357	48.78
Race/ethnicity		
Hispanic	11,136	22.55
Non-Hispanic white	55,235	51.58
Non-Hispanic black	8,073	13.43
Non-Hispanic mixed race	4,649	4.68
Other (Asians/Pacific Islanders and American Indians)	6,442	7.76
Household composition		
Two-parent biological	58,306	63.08
Two-parent stepfamily	6,517	9.64
Single mother	13,708	19.19
Other family type	7,004	8.10
Place of residence		
Metropolitan	62,845	84.35
Nonmetropolitan	21,486	15.65
Highest household or parental education level (years)		
<12	4,893	11.64
12	12,771	19.91
13–15	21,500	24.66
16+	44,186	43.79
Household poverty status (ratio of family income to poverty threshold)		
Below 100%	12,882	21.95
100–199%	15,347	21.72
200–399%	26,139	28.44
At or above 400%	31,167	27.90

**Table 3:** Weighted prevalence of selected mental health outcomes among US children aged 2–17 years by perinatal and sociodemographic characteristics: the 2011-2012 National Survey of Children's Health (*N* = 84,685).

Sociodemographic characteristics	Emotional/behavioral problem			Serious emotional/ behavioral problem			Depression			Anxiety			Oppositional defiant or conduct disorder		
	%	SE	*P* value	%	SE	*P* value	%	SE	*P* value	%	SE	*P* value	%	SE	*P* value
Child born prematurely			<0.001			<0.001			0.065			<0.001			<0.001
Premature	8.10	0.54		5.29	0.44		2.69	0.32		4.72	0.37		4.50	0.41	
Not premature	5.70	0.19		3.36	0.15		2.06	0.13		3.13	0.13		2.95	0.15	
Child's birthweight			0.086			0.293			0.555			0.242			0.044
Low birthweight	6.86	0.59		3.99	0.42		2.33	0.39		3.86	0.42		4.12	1.10	
Very low birthweight	7.30	1.31		5.01	1.21		2.12	0.60		4.05	0.81		4.12	1.10	
Moderately low birthweight	6.77	0.66		3.78	0.44		2.37	0.46		3.76	0.48		3.81	0.46	
Normal birthweight	5.80	0.20		3.52	0.16		2.08	0.13		3.29	0.14		2.96	0.15	
Child's age (years)			<0.001			<0.001			<0.001			<0.001			<0.001
2–5	2.03	0.23		1.22	0.17		0.26	0.08		0.83	0.14		1.50	0.20	
6–11	6.17	0.30		3.87	0.25		1.49	0.16		3.50	0.20		3.81	0.25	
12–17	8.46	0.35		4.95	0.26		4.00	0.26		4.81	0.25		3.65	0.24	
Child's sex			<0.001			<0.001			0.252			0.014			<0.001
Male	7.12	0.27		4.48	0.23		2.28	0.17		3.65	0.18		4.34	0.23	
Female	4.90	0.24		2.75	0.17		2.01	0.16		3.03	0.17		1.97	0.16	
Race/ethnicity			<0.001			<0.001			0.004			<0.001			<0.001
Hispanic	4.79	0.46		3.04	0.38		1.73	0.28		1.98	0.24		3.03	0.39	
Non-Hispanic white	6.79	0.24		4.01	0.19		2.32	0.16		4.37	0.2		3.07	0.17	
Non-Hispanic black	6.10	0.47		3.74	0.36		2.25	0.3		2.33	0.31		4.37	0.39	
Non-Hispanic mixed race	8.11	0.79		5.41	0.68		3.53	0.6		4.26	0.53		4.67	0.62	
Other	3.33	0.44		1.64	0.23		1.27	0.34		1.78	0.25		1.47	0.24	
Household composition			<0.001			<0.001			<0.001			<0.001			<0.001
Two-parent biological	3.83	0.18		2.10	0.14		0.97	0.09		2.51	0.14		1.65	0.13	
Two-parent stepfamily	9.99	0.82		6.04	0.65		4.66	0.62		4.98	0.52		5.95	0.72	
Single mother	9.74	0.50		6.43	0.42		3.97	0.34		4.92	0.34		5.63	0.39	
Other family type	9.82	0.85		6.14	0.67		4.08	0.62		4.22	0.48		6.05	0.64	
Place of residence			<0.001			<0.001			<0.001			<0.001			0.032
Metropolitan	6.04	0.21		3.63	0.16		2.08	0.13		3.37	0.14		3.19	0.16	
Non-metropolitan	6.56	0.38		4.08	0.31		2.77	0.28		3.59	0.28		3.39	0.24	
Highest household/parental education (years)			<0.001			<0.001			<0.001			<0.001			<0.001
<12	6.83	0.69		4.18	0.49		2.75	0.43		2.35	0.33		4.39	0.55	
12	7.11	0.43		4.30	0.34		2.79	0.28		3.01	0.24		4.72	0.37	
13–15	7.84	0.43		5.07	0.36		3.00	0.29		4.42	0.32		4.16	0.31	
16+	4.54	0.23		2.51	0.18		1.33	0.14		3.29	0.19		1.73	0.17	
Household poverty status			<0.001			<0.001			<0.001			0.064			<0.001
Below 100%	9.05	0.50		5.82	0.40		3.48	0.32		3.83	0.28		5.94	0.43	
100–199%	6.66	0.41		4.01	0.31		2.50	0.25		3.65	0.31		3.68	0.29	
200–399%	5.18	0.36		3.15	0.30		1.95	0.24		2.99	0.24		2.52	0.27	
At or above 400%	4.06	0.24		2.13	0.17		1.03	0.13		3.11	0.21		1.32	0.16	

*P* values associated with chi-square tests for independence between each covariate and mental health outcome.

**Table 4:** Weighted prevalence of selected mental-health-related conditions among US children aged 2–17 years by perinatal and sociodemographic characteristics: the 2011-2012 National Survey of Children's Health (*N* = 84,685).

Sociodemographic characteristics	Autism spectrum disorder			ADD/ADHD			Developmental delay			Learning disability			Intellectual disability/mental retardation		
	%	SE	*P* value	%	SE	*P* value	%	SE	*P* value	%	SE	*P* value	%	SE	*P* value
Child born prematurely			<0.001			<0.001			<0.001			<0.001			<0.001
Premature	3.57	0.46		10.51	0.63		8.29	0.58		13.86	0.76		2.35	0.29	
Not premature	1.67	0.11		7.44	0.22		2.91	0.13		7.18	0.22		0.87	0.08	
Child's birthweight			0.053			0.201			<0.001			<0.001			<0.001
Low birthweight	2.67	0.42		8.52	0.60		7.53	0.60		12.84	0.81		2.25	0.34	
Very low birthweight	4.50	1.75		10.90	1.81		14.56	1.99		18.16	2.38		3.93	1.39	
Moderately low birthweight	2.28	0.34		8.01	0.62		6.02	0.59		11.68	0.85		1.89	0.29	
Normal birthweight	1.83	0.12		7.72	0.22		3.06	0.14		7.37	0.22		0.89	0.08	
Child's age (years)			0.095			<0.001			0.002			<0.001			<0.001
2–5	1.49	0.22		1.49	0.16		3.97	0.31		3.30	0.30		0.47	0.08	
6–11	2.14	0.19		9.19	0.37		3.87	0.23		8.16	0.35		1.11	0.14	
12–17	1.86	0.15		10.69	0.37		2.99	0.19		10.20	0.36		1.38	0.15	
Child's sex			<0.001			<0.001			<0.001			<0.001			<0.001
Male	3.04	0.20		10.73	0.33		4.63	0.21		10.04	0.32		1.33	0.13	
Female	0.69	0.08		4.91	0.24		2.43	0.16		5.87	0.27		0.78	0.09	
Race/ethnicity			<0.001			<0.001			<0.001			<0.001			0.006
Hispanic	1.57	0.31		4.83	0.46		2.69	0.32		7.19	0.55		0.94	0.23	
Non-Hispanic white	2.23	0.13		9.45	0.29		3.77	0.17		8.30	0.27		1.08	0.1	
Non-Hispanic black	1.46	0.25		8.73	0.60		4.53	0.44		9.62	0.63		1.39	0.22	
Non-Hispanic mixed race	2.55	0.41		9.53	0.8		3.99	0.48		8.73	0.77		1.25	0.28	
Other	1.01	0.18		3.91	0.41		2.73	0.35		5.16	0.52		0.58	0.13	
Household composition			0.459			<0.001			<0.001			<0.001			0.009
Two-parent biological	1.80	0.12		5.76	0.22		3.10	0.16		6.45	0.23		0.84	0.09	
Two-parent stepfamily	1.99	0.44		13.77	0.95		3.64	0.40		9.40	0.78		1.09	0.21	
Single mother	2.21	0.24		10.85	0.54		4.48	0.32		11.17	0.57		1.38	0.19	
Other family type	1.78	0.48		10.46	0.72		4.83	0.59		10.64	0.86		1.98	0.49	
Place of residence			<0.001			0.005			0.919			0.398			0.607
Metropolitan	2.00	0.13		7.69	0.23		3.55	0.15		7.97	0.24		1.06	0.09	
Non-metropolitan	1.55	0.16		9.22	0.45		3.61	0.27		8.43	0.45		1.10	0.14	
Highest household/parental education (years)			<0.001			<0.001			0.003			<0.001			0.007
<12	1.10	0.29		6.31	0.58		3.20	0.43		9.8	0.82		1.53	0.35	
12	1.36	0.16		9.87	0.53		3.95	0.30		10.65	0.57		1.26	0.17	
13–15	2.22	0.26		9.13	0.44		4.35	0.33		9.36	0.47		1.20	0.20	
16+	2.28	0.18		6.88	0.29		3.03	0.17		5.69	0.24		0.75	0.08	
Household poverty status			0.038			<0.001			<0.001			<0.001			<0.001
Below 100%	1.45	0.16		9.47	0.51		4.64	0.33		12.12	0.61		1.53	0.21	
100–199%	1.91	0.22		8.31	0.46		3.91	0.28		7.89	0.43		1.08	0.15	
200–399%	2.31	0.26		7.47	0.39		3.43	0.29		7.42	0.42		1.04	0.19	
At or above 400%	1.80	0.16		6.74	0.33		2.55	0.19		5.55	0.27		0.69	0.08	

*P* values associated with chi-square tests for independence between each covariate and mental health outcome.

**Table 5:** Adjusted odds ratios (AOR) and 95% confidence intervals for selected mental health outcomes among US children aged 2–17 years by perinatal and sociodemographic characteristics: the 2011-2012 National Survey of Children's Health (*N* = 84,685).

Sociodemographic characteristics	Emotional/behavioral problem		Serious emotional/behavioral problem		Depression		Anxiety		Oppositional defiant or conduct disorder	
	AOR	95% CI	AOR	95% CI	AOR	95% CI	AOR	95% CI	AOR	95% CI
Child born prematurely										
Premature	1.47	1.25–1.73	1.61	1.32–1.96	1.33	1.01–1.74	1.58	1.31–1.91	1.50	1.21–1.86
Not premature^1^	1.00		1.00		1.00		1.00		1.00	
Child's birthweight										
Low birthweight	1.25	1.03–1.52	1.18	0.93–1.49	1.13	0.79–1.62	1.32	1.04–1.68	1.28	1.00–1.63
Very low birthweight	1.31	0.88–1.96	1.47	0.87–2.48	0.98	0.54–1.80	1.49	0.97–2.30	1.26	0.73–2.19
Moderately low birthweight	1.24	0.99–1.54	1.12	0.86–1.44	1.16	0.77–1.74	1.29	0.98–1.70	1.28	0.98–1.67
Normal birthweight^1^	1.00		1.00		1.00		1.00		1.00	
Child's age (years)										
2–5^1^	1.00		1.00		1.00		1.00		1.00	
6–11	3.09	2.41–3.96	3.14	2.31–4.28	5.44	2.95–10.02	4.22	2.99–5.95	2.49	1.85–3.34
12–17	4.23	3.33–5.39	3.95	2.92–5.34	14.44	8.03–25.97	5.68	4.04–7.98	2.28	1.70–3.06
Child's sex										
Male	1.50	1.31–1.72	1.68	1.42–1.99	1.12	0.90–1.40	1.20	1.03–1.41	2.33	1.90–2.87
Female^1^	1.00		1.00		1.00		1.00		1.00	
Race/ethnicity										
Non-Hispanic white^1^	1.00		1.00		1.00		1.00		1.00	
Hispanic	0.54	0.42–0.68	0.58	0.42–0.81	0.60	0.40–0.89	0.42	0.32–0.57	0.66	0.48–0.92
Non-Hispanic black	0.50	0.41–0.61	0.49	0.38–0.62	0.48	0.35–0.67	0.33	0.24–0.44	0.74	0.59–0.93
Non-Hispanic mixed race	0.97	0.77–1.22	1.06	0.79–1.41	1.21	0.82–1.79	0.83	0.62–1.10	1.16	0.85–1.57
Other	0.48	0.35–0.66	0.42	0.30–0.60	0.73	0.41–1.28	0.42	0.30–0.59	0.42	0.28–0.64
Household composition										
Two-parent biological^1^	1.00		1.00		1.00		1.00		1.00	
Two-parent stepfamily	2.16	1.74–2.68	2.27	1.72–3.00	3.44	2.39–4.95	1.70	1.32–2.18	2.80	2.02–3.89
Single mother	2.26	1.91–2.68	2.60	2.09–3.23	3.25	2.42–4.35	2.08	1.69–2.57	2.42	1.92–3.07
Other family type	2.46	1.95–3.08	2.71	2.03–3.60	3.48	2.38–5.09	1.81	1.38–2.36	3.09	2.29–4.18
Place of residence										
Metropolitan	1.20	1.04–1.39	1.17	0.97–1.40	1.03	0.81–1.32	1.19	0.99–1.44	1.28	1.06–1.54
Non-metropolitan^1^	1.00		1.00		1.00		1.00		1.00	
Highest household/parental education (years)										
<12	0.93	0.70–1.25	0.91	0.64–1.31	1.08	0.68–1.71	0.56	0.38–0.81	1.20	0.81–1.78
12	0.99	0.81–1.21	0.99	0.76–1.29	1.11	0.79–1.56	0.66	0.52–0.84	1.38	1.02–1.88
13–15	1.25	1.04–1.50	1.36	1.07–1.72	1.36	0.98–1.89	1.08	0.87–1.35	1.43	1.07–1.90
16+^1^	1.00		1.00		1.00		1.00		1.00	
Household poverty status										
Below 100%	2.50	2.00–3.12	2.76	2.05–3.72	2.96	2.10–4.17	1.95	1.49–2.55	3.56	2.51–5.06
100–199%	1.63	1.32–2.01	1.72	1.32–2.26	2.01	1.42–2.84	1.46	1.13–1.88	2.21	1.57–3.09
200–399%	1.21	1.01–1.48	1.38	1.04–1.82	1.64	1.16–2.32	0.99	0.79–1.25	1.66	1.17–2.35
At or above 400%^1^	1.00		1.00		1.00		1.00		1.00	

The effects of prematurity and birthweight were estimated by separate logistic regression models that controlled for age, sex, race/ethnicity, household composition, place of residence, and household education and income levels. ^1^Reference group.

**Table 6:** Adjusted odds ratios (AOR) and 95% confidence intervals for selected mental-health-related conditions among US children aged 2–17 years by perinatal and sociodemographic characteristics: the 2011-2012 National Survey of Children's Health (*N* = 84,685).

Sociodemographic characteristics	Autism spectrum disorder		ADD/ADHD		Developmental delay		Learning disability		Intellectual disability/ mental retardation	
	AOR	95% CI	AOR	95% CI	AOR	95% CI	AOR	95% CI	AOR	95% CI
Child born prematurely										
Premature	2.26	1.69–3.03	1.49	1.29–1.73	2.92	2.44–3.49	2.13	1.84–2.46	2.74	2.02–3.73
Not premature^1^	1.00		1.00		1.00		1.00		1.00	
Child's birthweight										
Low birthweight	1.80	1.28–2.53	1.23	1.04–1.46	2.64	2.18–3.20	1.95	1.66–2.30	2.62	1.84–3.72
Very low birthweight	3.16	1.42–7.04	1.67	1.13–2.47	5.43	3.93–7.50	2.88	2.04–4.06	4.40	2.07–9.36
Moderately low birthweight	1.53	1.10–2.11	1.14	0.95–1.37	2.09	1.67–2.62	1.76	1.47–2.11	2.23	1.58–3.15
Normal birthweight^1^	1.00		1.00		1.00		1.00		1.00	
Child's age (years)										
2–5^1^	1.00		1.00		1.00		1.00		1.00	
6–11	1.49	1.05–2.12	6.59	5.20–8.35	0.98	0.80–1.20	2.67	2.17–3.29	2.38	1.57–3.59
12–17	1.30	0.93–1.83	7.49	5.92–9.48	0.75	0.60–0.92	3.46	2.82–4.23	2.97	1.97–4.48
Child's sex										
Male	4.49	3.48–5.80	2.43	2.15–2.75	1.96	1.66–2.30	1.82	1.61–2.05	1.70	1.25–2.31
Female^1^	1.00		1.00		1.00		1.00		1.00	
Race/ethnicity										
Non-Hispanic white^1^	1.00		1.00		1.00		1.00		1.00	
Hispanic	0.85	0.53–1.36	0.42	0.33–0.54	0.60	0.44–0.81	0.65	0.53–0.80	0.65	0.36–1.19
Non-Hispanic black	0.61	0.41–0.92	0.64	0.53–0.77	0.90	0.71–1.15	0.80	0.67–0.94	0.87	0.60–1.26
Non-Hispanic mixed race	1.07	0.75–1.55	0.91	0.74–1.11	0.89	0.68–1.17	0.93	0.76–1.15	1.00	0.61–1.64
Other	0.60	0.40–0.89	0.33	0.25–0.43	0.60	0.43–0.83	0.54	0.41–0.71	0.41	0.23–0.76
Household composition										
Two-parent biological^1^	1.00		1.00		1.00		1.00		1.00	
Two-parent stepfamily	1.16	0.71–1.89	1.99	1.65–2.39	1.11	0.87–1.42	1.13	0.92–1.39	0.99	0.64–1.55
Single mother	1.53	1.15–2.04	1.79	1.54–2.08	1.23	1.00–1.51	1.36	1.17–1.58	1.19	0.78–1.83
Other family type	1.21	0.67–2.18	1.66	1.39–1.99	1.52	1.12–2.08	1.40	1.13–1.74	1.79	0.91–3.52
Place of residence										
Metropolitan	1.43	1.13–1.83	1.02	0.90–1.16	1.16	0.97–1.38	1.17	1.02–1.33	1.17	0.86–1.58
Non-metropolitan^1^	1.00		1.00		1.00		1.00		1.00	
Highest household/parental education (years)										
<12	0.44	0.24–0.81	0.81	0.62–1.06	0.81	0.58–1.15	1.35	1.07–1.72	1.59	0.90–2.81
12	0.52	0.38–0.71	1.17	0.98–1.38	0.97	0.77–1.21	1.53	1.28–1.84	1.30	0.87–1.96
13–15	0.86	0.63–1.18	1.11	0.95–1.30	1.16	0.93–1.45	1.44	1.22–1.68	1.32	0.84–2.06
16+^1^	1.00		1.00		1.00		1.00		1.00	
Household poverty status										
Below 100%	1.19	0.79–1.77	1.68	1.38–2.05	2.00	1.54–2.61	2.04	1.68–2.48	1.93	1.16–3.22
100–199%	1.41	1.01–1.97	1.27	1.06–1.52	1.59	1.24–2.03	1.24	1.04–1.48	1.37	0.90–2.07
200–399%	1.50	1.11–2.01	1.08	0.92–1.28	1.33	1.06–1.68	1.27	1.08–1.50	1.43	0.95–2.15
At or above 400%^1^	1.00		1.00		1.00		1.00		1.00	

The effects of prematurity and birthweight were estimated by separate logistic regression models that controlled for age, sex, race/ethnicity, household composition, place of residence, and household education and income levels. ^1^Reference group.

**Table 7:** Sex differentials in adjusted odds of selected mental health outcomes among US children and adolescents according to premature birth and low birthweight status: the 2011-2012 National Survey of Children's Health (*N* = 84,685).

Mental health outcome	Adjusted odds for prematurity		Adjusted odds for LBW versus normal birthweight		Adjusted odds for VLBW versus normal birthweight		Adjusted odds for MLBW versus normal birthweight	
	AOR^a^	95% CI	AOR^a^	95% CI	AOR^a^	95% CI	AOR^a^	95% CI
Males								
Behavioral/emotional problem	1.48	1.20–1.82	1.04	0.81–1.33	1.29	0.72–2.31	0.98	0.75–1.29
Serious emotional health problem	1.50	1.18–1.91	0.96	0.71–1.31	1.38	0.63–3.01	0.87	0.65–1.18
Depression	1.35	0.95–1.90	0.92	0.62–1.38	0.95	0.39–2.33	0.92	0.60–1.42
Anxiety	1.59	1.24–2.02	1.02	0.76–1.37	1.06	0.57–1.99	1.02	0.74–1.40
Conduct problem	1.49	1.14–1.95	1.03	0.74–1.43	1.21	0.56–2.62	0.99	0.69–1.41
Autism spectrum disorder	2.18	1.54–3.08	1.49	0.96–2.31	3.41	1.35–8.61	1.09	0.75–1.60
ADD/ADHD	1.51	1.27–1.81	1.21	0.97–1.50	1.48	0.83–2.61	1.15	0.92–1.45
Developmental delay	2.65	2.10–3.33	2.29	1.77–2.95	4.44	2.75–7.17	1.85	1.38–2.46
Learning disability	1.94	1.62–2.33	1.73	1.41–2.14	2.30	1.42–3.73	1.61	1.29–2.03
Intellectual disability	2.58	1.73–3.85	2.10	1.24–3.54	4.77	1.66–13.71	1.53	1.01–2.31
Females								
Behavioral/emotional problem	1.47	1.14–1.91	1.53	1.14–2.05	1.36	0.80–2.31	1.56	1.12–2.17
Serious emotional health problem	1.79	1.28–2.49	1.47	1.02–2.10	1.62	0.85–3.09	1.44	0.97–2.15
Depression	1.29	0.83–2.00	1.32	0.77–2.25	1.04	0.46–2.34	1.38	0.76–2.52
Anxiety	1.58	1.18–2.12	1.64	1.14–2.35	1.99	1.10–3.59	1.57	1.04–2.37
Conduct problem	1.51	1.05–2.16	1.78	1.23–2.59	1.39	0.72–2.67	1.87	1.25–2.81
Autism spectrum disorder	2.54	1.54–4.21	2.97	1.73–5.10	2.18	0.99–4.78	3.11	1.73–5.59
ADD/ADHD	1.43	1.11–1.84	1.26	0.97–1.64	2.04	1.27–3.26	1.12	0.82–1.51
Developmental delay	3.50	2.64–4.64	3.25	2.40–4.41	7.41	4.83–11.38	2.51	1.74–3.62
Learning disability	2.45	1.94–3.11	2.25	1.75–2.89	3.56	2.19–5.80	1.98	1.50–2.62
Intellectual disability	3.00	1.87–4.82	3.53	2.17–5.73	3.82	1.88–7.75	3.47	2.00–6.00

^a^Adjusted by logistic regression for child's age, race/ethnicity, household composition, metro/nonmetro residence, household poverty, and education levels. LBW: low birthweight. VLBW: very low birthweight. MLBW: moderately low birthweight.
